# Serial circulating tumour DNA analysis for locally advanced rectal cancer treated with preoperative therapy: prediction of pathological response and postoperative recurrence

**DOI:** 10.1038/s41416-020-0941-4

**Published:** 2020-06-22

**Authors:** Satoshi Murahashi, Takashi Akiyoshi, Takeshi Sano, Yosuke Fukunaga, Tetsuo Noda, Masashi Ueno, Hitoshi Zembutsu

**Affiliations:** 1 Department of Gastrointestinal Surgery, Cancer Institute Hospital, Japanese Foundation for Cancer Research, Tokyo, Japan; 2 Cancer Institute, Japanese Foundation for Cancer Research, Tokyo, Japan; 3 Project for Development of Liquid Biopsy Diagnosis, Cancer Precision Medicine Center, Japanese Foundation for Cancer Research, Tokyo, Japan

**Keywords:** Rectal cancer, Tumour biomarkers

## Abstract

**Background:**

The “watch-and-wait” approach is a common treatment option amongst patients with locally advanced rectal cancer (LARC). However, the diagnostic sensitivity of clinical modalities, such as colonoscopy and magnetic resonance imaging to determine pathological response, is not high. We analysed the clinical utility of circulating tumour DNA (ctDNA) of patients with LARC to predict response to preoperative therapy and postoperative recurrence.

**Methods:**

A serial ctDNA analysis of 222 plasma samples from 85 patients with LARC was performed using amplicon-based deep sequencing on a cell-free DNA panel covering 14 genes with over 240 hotspots.

**Results:**

ctDNA was detected in 57.6% and 22.3% of samples at baseline and after preoperative treatment, respectively, which was significantly different (*P* = 0.0003). Change in ctDNA was an independent predictor of complete response to preoperative therapy (*P* = 0.0276). In addition, postoperative ctDNA and carcinoembryonic antigen (CEA) were independent prognostic markers for risk of recurrence after surgery (ctDNA, *P* = 0.0127 and CEA, *P* = 0.0105), with a combined analysis having cumulative effects on recurrence-free survival (*P* = 1.0 × 10^–16^).

**Conclusions:**

Serial ctDNA analysis may offer clinically useful predictive and prognostic markers for response to preoperative therapy and postoperative recurrence in patients with LARC.

## Background

Rectal cancer is the second-most common cancer of the large intestine, and a major health burden globally. Radiation therapy can improve local recurrence rates in patients with locally advanced rectal cancer (LARC),^1–3^ with preoperative chemoradiation therapy (CRT) considered the standard of care for these patients. Indeed, studies have shown that CRT before surgery can reduce tumour volume and invoke pathological complete response (pCR, ypT0N0M0) in 15–20% of patients.^4–6^ The achievement of pCR is associated with improved local and distant control, disease-free survival and overall survival; some patients who achieve pCR may not even require surgery.^7^ Although several studies have reported factors that are predictive of pCR after preoperative CRT,^8–11^ the utility of such factors to predict pCR in the clinical setting has not been identified. In addition, despite the range of options available to detect pCR, including digital rectal examinations, endoscopic assessments of mucosal integrity and magnetic resonance imaging (MRI) for changes in primary lesions, the accuracy of these modalities is questionable.^12–14^

The advent of liquid biopsies was expected to allow for an interrogation of the cancer genome while providing a minimally invasive approach for patients with cancer.^15^ Liquid biopsies are based on the analysis of circulating tumour DNA (ctDNA), which is secreted from cancer cells into the peripheral blood as a result of cell apoptosis and/or necrosis.^15,16^ However, ctDNA represents only a small fraction of the cell-free DNA (cfDNA), meaning that there is very little ctDNA with each blood sample drawn. Despite this limitation, various studies have reported the clinical utility of cfDNA and ctDNA for the treatment of colorectal cancer, in terms of monitoring treatment response, cancer recurrence and drug resistance.^17–20^ The clinical utility of ctDNA to predict patient responses to preoperative therapy, including CRT among patients with LARC, has not yet been reported.

A diagnostic system that could predict patient responses to preoperative therapy would help to select those patients with LARC who would be expected to achieve pCR, and provide the option for these patients to be managed without immediate surgery (i.e., the “watch-and-wait” approach).^7,21^ Thus, the aim of the present study was to investigate the clinical utility of serial ctDNA analysis to predict responses to preoperative therapy and clinical outcomes after surgical intervention in patients with LARC. To this end, we examined mutant allele fractions of 14 genes that are frequently mutated in colorectal cancer using targeted next-generation sequencing (NGS). Our results may aid in the development of a diagnostic system for personalised preoperative therapy, and help to improve survival prognostication and the stratification of patients with LARC.

## Methods

### Patients

This study prospectively enrolled patients with LARC between February 2017 and November 2018. All patients were diagnosed as clinical stage II or III (cT3-4N0, or cTanyN+) using the Union for International Cancer Control (UICC) TNM classification, and received preoperative therapy at the Cancer Institute Hospital, Japanese Foundation for Cancer Research. Thirty-three patients received standard CRT, comprising a total dose of 50.4 Gy in 28 fractions over 5 weeks and concurrent Tegafur/Gimeracil/Oteracil (80–120 mg/m^2^/day) orally administered over 4 weeks. Nine patients received short-course radiotherapy (SRT), with 25 Gy administered in 5 fractions over 1 week. Four patients with upper rectal cancer diagnosed as cT4 or cN2 received neoadjuvant chemotherapy (NAC), consisting of four cycles of capecitabine (2000 mg/m^2^/day), oxaliplatin (130 mg/m^2^) and bevacizumab (7.5 mg/kg) (CapeOx + Bmab). Twenty-three patients with lower rectal cancer diagnosed as cT4 or cN2 received a combination of 6 cycles of fluorouracil (bolus, 400 mg/m^2^; infusion, 2400 mg/m^2^), leucovorin (200 mg/m^2^), oxaliplatin (85 mg/m^2^) and bevacizumab (5 mg/kg) (FOLFOX + Bmab) as NAC, followed by CRT. In addition, 16 patients with cT3N1M0 and an intermediate risk of recurrence received a combination of SRT, followed by four cycles of CapeOx. All patients were histologically diagnosed with adenocarcinoma, and received pre-treatment rectal MRI and chest/abdomen/pelvis CT. All patients had an Eastern Cooperative Oncology Group performance status of 0–2, and received preoperative therapy followed by surgery or non-operative management. The decision for non-operative management was carefully discussed with each patient who achieved clinical complete response (cCR), according to the National Comprehensive Cancer Network (NCCN) guidelines. Patients with previous cancers were excluded. At 4–8 weeks after completing all neoadjuvant, preoperative therapies, all patients, except those receiving SRT, were re-staged using colonoscopy, rectal MRI and chest/abdomen/pelvis CT scan. The median time to surgery in patients receiving SRT was 10 days (IQR, 7–30 days): six patients receiving SRT underwent surgery within 15 days after SRT, but surgery was delayed for about 4 weeks after SRT in three patients. Endoscopic response was classified by mucosal findings, as reported by Chino et al.^22^ Clinicopathological factors were evaluated by UICC TNM classification. cCR was defined as ycT0N0M0, and pathological complete response (pCR) as ypT0N0M0. Dworak’s criteria were used for tumour regression grade (TRG): TRG 1, minimal regression; TRG 2, moderate regression; TRG 3, near-complete regression; TRG 4, complete regression.^23^ This study was approved by the Institutional Review Boards of the Japanese Foundation for Cancer Research (Tokyo, Japan). Written informed consent was obtained from all patients.

### Blood sampling, cell-free DNA isolation and sequencing

For ctDNA analysis, blood samples were collected before the initiation of preoperative therapy (baseline), after preoperative treatment (post treatment, just before surgery) and at 12 weeks after surgery (post operation). A total of 222 blood samples from 85 patients were collected into EDTA tubes following the manufacturer’s instructions.^15^ Plasma was extracted by centrifugation at 1600 × *g* for 10 min at 4 °C, followed by 16,000×*g* for 10 min at 4 °C to remove cellular debris. cfDNA was extracted from plasma using a MagMAX cfDNA Isolation Kit (Thermo Fisher Scientific), following the manufacturer’s instructions.^15^ cfDNA quality was checked using Qubit2.0 and 2100 Bioanalyzer (Agilent Technologies). Libraries from the cfDNA were prepared with Oncomine Colon cfDNA Assay (Thermo Fisher Scientific), and checked using Qubit2.0 and 2100 Bioanalyzer.^15^ Molecular barcoded amplicon-based deep sequencing was used in this assay to reduce the false positives derived from polymerase chain reaction (PCR) errors. The Ion Chef System and Ion 530 Kit-Chef were used for template preparation. Ion 530 chips were sequenced on an Ion S5 system. The six-plex library pool was applied to one Ion 530 chip. The cfDNA panel used in this study covered 14 genes (*TP53, KRAS, APC, PIK3CA, FBXW7, NRAS, GNAS, SMAD4, MAP2K1, ERBB2, BRAF, AKT1, CTNNB1* and *EGFR*) with more than 240 hotspots (SNVs and short indels). The clean reads were mapped to the human reference genome (hg19). Variant caller was used to filter and call mutations in the targeted regions of each gene.^24,25^ The cut-off value for the mutant allele fraction (MAF) was 0.15%. The average coverage ranged from 20,000 to 50,000. The total MAF of the detected mutant alleles in each patient was used as the metric for ctDNA in this study.

We also measured carcinoembryonic antigen (CEA) in the hospital central laboratory before preoperative therapy, after preoperative treatment and at 12 weeks after surgery.

### Statistical analysis

Continuous variables are expressed as the median and interquartile range (IQR). Fisher’s exact test was used to compare the proportion of patients with plasma mutations before and after preoperative therapy. Wilcoxon matched-pair signed-rank test was used to evaluate the significance of changes in ctDNA after preoperative therapy. Logistic regression analysis was used to identify significant predictive factors, and to test for the independent contribution of factors in response to preoperative therapy. To assess for potential confounding factors, gender, pre-treatment CEA, distance from the anal verge, clinical T and N factors, NAC with CRT/SRT and change in ctDNA were treated as categorical variables. Furthermore, Cox proportional hazard analysis was used to identify significant prognostic factors, and to test for an independent contribution of these factors to recurrence-free survival. In this case, potential confounding was assessed using gender, postoperative CEA, pathological T and N factors, lymphovascular invasion, response to preoperative therapy (Dworak’s TRG) and NAC with CRT/SRT. Postoperative ctDNA was treated as a categorical variable. Variables with a *P* value less than 0.2 in the univariate analysis were included in the multivariate analysis. Recurrence-free survival curves were visualised by the Kaplan–Meier method. The differences between the curves were estimated using the log-rank test. Logistic regression and Cox proportional hazard analyses were performed using JMP statistical software. Other analyses were performed using GraphPad Prism7. All *P* values were two-sided. Values with *P* < 0.05 were considered to be statistically significant. We used a significance level of 0.0025 (0.05/20 clinical factors) in the association study of clinical factors with ctDNA (Table 1) to adjust for multiple testing by Bonferroni correction.

**Table 1:** Patient characteristics.

		Baseline ctDNA (*n* = 82)		*P*	Post-treatment ctDNA (*n* = 81)		*P*	Postoperative ctDNA (*n* = 59)		*P*
		Positive (*n* = 49)	Negative (*n* = 33)		Positive (*n* = 19)	Negative (*n* = 62)		Positive (*n* = 21)	Negative (*n* = 38)	
Age, years										
Median	60	59	60	0.8822	66	60.5	0.4043	67	56	0.1038
IQR	52–69	52–70	52–70		51–69	52–70		51–70	51–69	
Gender, *n* (%)										
Male	65 (76.5)	38	25	>0.9999	14	49	0.7532	15	32	0.3155
Female	20 (23.5)	11	8		5	13		6	6	
Interval from preoperative therapy to surgery, days										
Median	54	55	52	0.5454	53	52	0.7886	52	55	0.8413
IQR	33–68.5	33–65.5	32.8–67.8		30.3–59.8	33–65		31.8–65	32.3–64.3	
Distance from anal verge (cm), *n* (%)										
≥4	59 (69.4)	38	20	0.1377	12	43	0.7794	16	25	0.5570
<4	26 (30.6)	11	13		7	19		5	13	
Clinical T factor, *n* (%)										
cT3	66 (77.6)	38	25	>0.9999	17	45	0.2148	16	28	>0.9999
cT4	19 (22.4)	11	8		2	17		5	10	
Pre-treatment CEA, *n* (%)										
Normal	47 (55.3)	24	22	0.1730	8	36	0.2942	10	18	>0.9999
High	38 (44.7)	25	11		11	26		11	20	
Post-treatment CEA, *n* (%)										
Normal	74 (87.1)	42	29	>0.9999	10	60	<0.0001	16	36	0.0853
High	11 (12.9)	7	4		9	2		5	2	
Postoperative CEA, *n* (%)										
Normal	52 (88.1)	30	19	>0.9999	10	42	0.0479	17	35	0.2333
High	7 (11.9)	4	3		4	3		4	3	
Clinical stage, *n* (%)										
II	27 (31.8)	14	13	0.3444	4	20	0.4048	5	15	0.2632
III	58 (68.2)	35	20		15	42		16	23	
Preoperative therapy, *n* (%)										
NAC with CRT/SRT	39 (45.9)	26	12	0.1770	13	25	0.0385	11	17	0.5981
Others	46 (54.1)	23	21		6	37		10	21	
Endoscopic response, *n* (%)										
Complete response	19 (22.4)	13	6	0.4340	1	14	0.1731	3	6	>0.9999
Incomplete response	66 (77.6)	36	27		18	48		18	32	
Clinical response, *n* (%)										
Complete response	12 (14.1)	8	4	0.7539	1	7	0.6732	1	2	>0.9999
Incomplete response	73 (85.9)	41	29		18	55		20	36	
Pathological T factor, *n* (%)										
ypT0–2	38 (44.7)	23	13	0.4858^a^	7	31	0.2914^a^	9	22	0.2911
ypT3–4	39 (45.9)	21	17		12	27		12	16	
NA (watch and wait)	8 (9.4)	5	3		0	4		NA	NA	
Pathological N factor, *n* (%)										
ypN0	51 (60.0)	29	22	0.6115^a^	13	38	>0.9999^a^	11	24	0.5806
ypN1–2	26 (30.6)	15	8		6	20		10	14	
NA (watch and wait)	8 (9.4)	5	3		0	4		NA	NA	
Lymphatic invasion, *n* (%)										
Negative	64 (75.3)	36	25	>0.9999^a^	15	49	0.7248^a^	18	31	0.9546
Positive	13 (15.3)	8	5		4	9		3	7	
NA (watch and wait)	8 (9.4)	5	3		0	4		NA	NA	
Venous invasion, *n* (%)										
Negative	54 (63.5)	29	22	0.6115^a^	9	45	0.0200^a^	14	29	0.5430
Positive	23 (27.1)	15	8		10	13		7	9	
NA (watch and wait)	8 (9.4)	5	3		0	4		NA	NA	
Response to preoperative therapy, *n* (%)										
pCR or ycCR (watch and wait)	21 (24.7)	13	8	>0.9999	2	15	0.3344	3	8	0.7301^a^
Non-pCR	64 (75.3)	36	25		17	47		18	30	
Histology, *n* (%)										
Well, mod	74 (87.0)	44	27	0.3362	18	53	0.4385	18	32	>0.9999
muc, por, sig	11 (13.0)	5	6		1	9		3	6	
Recurrence, *n* (%)										
Yes	11 (18.6)	8	3	0.4979	5	6	0.1096	5	6	0.4977
No	48 (81.4)	26	19		9	39		16	32	
Recurrence location, *n* (%)										
Lung^b^	8 (72.7)	6	2	>0.9999	2	6	0.0606	2	6	0.0606
Liver	3 (27.3)	2	0		3	0		3	0	

*CEA* carcinoembryonic antigen, *NAC* neoadjuvant chemotherapy, *CRT* chemoradiotherapy, *SRT* short-course radiotherapy, *pCR* pathological complete response, *cCR* clinical complete response, *NA* not analysed.

^a^Fisher's exact test was performed, excluding the patients followed up by watch-and-wait approach.

^b^One patient had synchronous bone metastasis.

## Results

### Clinicopathological characteristics

**Fig. 1:**
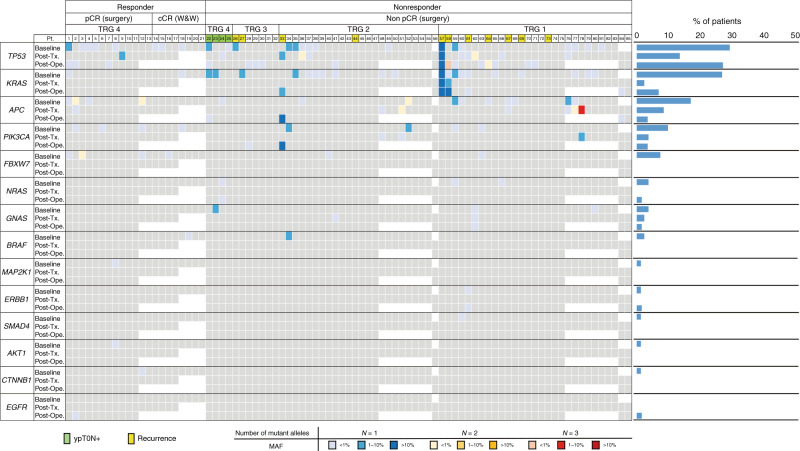
Genomic landscape of mutation detected in plasma. Gene mutations in 14 genes from samples retrieved from 85 patients with LARC. Grey, no mutation detected; white, analysis not conducted. pCR pathological complete response, cCR clinical complete response, TRG tumour regression grade (Dworak), W&W watch-and-wait, Post-Tx post–preoperative treatment, Post-Ope post operation, MAF the highest mutant allele fraction in each patient.

We prospectively recruited 85 patients with LARC who were receiving preoperative chemotherapy and/or RT. Table 1 shows the characteristics of these patients. At recruitment, the median age was 60 years (IQR, 52–69 years), 65 (76.5%) patients were male and 58 (68.2%) had been diagnosed as clinical stage III (cTanyN1–2). Thirty-nine (45.9%) patients received NAC with CRT/SRT (Table 1). After preoperative therapy, 12 (14.1%) patients achieved cCR (ycT0N0M0) (Table 1). Eight of these 12 patients chose non-operative management (“watch-and-wait” approach) after therapy (Table 1; Supplementary Fig. 1). The remaining 77 (90.6%) patients of the original cohort were treated surgically (Table 1; Supplementary Fig. 1), of which 17 (22.1%) patients were diagnosed as Dworak’s TRG 4 (Fig. 1). Of these 17 patients, four patients had an absence of cancer cells at the primary lesion, but were positive for pathological lymph node metastasis (ypT0N+). The other 13 patients achieved pCR (ypT0N0M0) after preoperative therapy (Fig. 1). The median interval from completing preoperative therapy to surgery was 54 days (IQR, 33–68.5, Table 1).

### Detection of somatic mutations in plasma

**Fig. 2:**
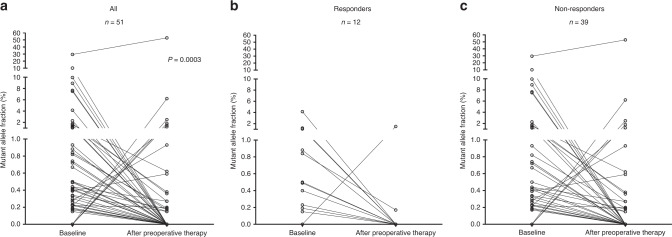
Change in circulating tumour DNA after preoperative therapy. Mutant allele fractions were significantly decreased after preoperative therapy in all patients (**a**), responders (**b**) and non-responders (**c**).

A total of 222 plasma samples from 85 patients were analysed by amplicon-based deep sequencing. One or more somatic mutations (mutant alleles) were detected in 49 (57.6%) patients at baseline (before preoperative therapy), but only in 19 (22.3%) patients after preoperative treatment, which was a significant reduction (*P* < 0.0001, Supplementary Fig. 2). No significant associations were observed between baseline or postoperative ctDNA status and any clinicopathological factors (Table 1). In contrast, post-treatment ctDNA detection was associated with post-treatment and postoperative CEA level, preoperative therapy regimens of NAC with CRT/SRT and venous invasion (Table 1). Only post-treatment CEA was significantly associated with ctDNA after Bonferroni correction (*P* < 0.0001). Mutations in *TP53*, *KRAS* and *APC* genes were detected in 24 (29.3%), 22 (26.8%) and 14 (17.1%) patients at baseline, respectively (Fig. 1 and Supplementary Fig. 3). Mutations in other genes were less common (<10% of patients) (Fig. 1 and Supplementary Figs. 3 and 4). Thirty-two (68.1%) mutations detected at baseline were not detected after preoperative therapy (Fig. 2). *TP53* (13 mutations) and *APC* (11 mutations) were the most frequently mutated genes after preoperative therapy (Fig. 1; Supplementary Fig. 4).

### Association between response to preoperative therapy and ctDNA response

Patients were classified into two groups in this study: responders (*n* = 21) and non-responders (*n* = 64). Responders were patients who achieved pCR after preoperative therapy or who were managed by the watch-and-wait approach for more than 12 months after achieving cCR (ycT0N0M0). Non-responders were patients who did not achieve pCR after preoperative therapy. No significant association was observed between baseline, post-treatment and postoperative ctDNA status and the rate of responders (Table 1).

To examine the change in MAFs in response to preoperative treatment, we used data from 51 patients (12 responders and 39 non-responders) for whom mutation(s) were detected before and/or after preoperative treatment (Fig. 2). MAF after preoperative treatment (median, 0%; IQR, 0–0.27%) was significantly lower than that at baseline (median, 0.49%; IQR, 0.23–1.22%; *P* = 0.0003), as shown in Fig. 2. Of the 12 responders, all but one patient showed a decrease in MAFs after preoperative treatment (Fig. 2b). Post-treatment ctDNA was detected in this one responder (8.3%), who had no detectable ctDNA at baseline. In comparison, 7 of the 39 non-responders showed an increase in MAF (Fig. 2c). Post-treatment ctDNA was detected in the three non-responders (7.7%) for whom there was also no detectable ctDNA at baseline. We performed a univariate logistic regression analysis to identify which factors were associated with response to preoperative therapy. We found a significant association between response and change in ctDNA (≥80% vs < 80%, *P* = 0.015; OR, 8.5; 95% CI, 1.4–163, Table 2). Preoperative therapy regimens of NAC with CRT/SRT showed a trend towards an increased response to preoperative therapy (*P* = 0.1178; OR, 2.9; 95% CI, 0.8–12.3, Table 2). In the multivariate analysis, the change in ctDNA still remained an independent predictor for response to preoperative therapy after adjustment (*P* = 0.0276; adjusted OR, 7.4; 95% CI, 1.2–144 for the change in ctDNA, Table 2).

**Table 2:** Logistic regression analysis for the response to preoperative therapy (pCR).

Variables	Univariate analysis		Multivariate analysis	
	OR (95% CI)	*P* value	OR (95% CI)	*P* value
Gender: male vs female	1.7 (0.4–12)	0.5099		
Pre-treatment CEA: < 5.0 ng/ml vs ≥5.0 ng/ml	1.1 (0.3–3.9)	0.9381		
Distance from anal verge: ≥4 cm vs < 4 cm	2.0 (0.4–14)	0.4072		
Clinical T factor: cT3 vs cT4	0.8 (0.2–4.1)	0.7441		
Clinical N factor: negative vs positive	1.3 (0.3–5.0)	0.7353		
NAC with CRT/SRT: yes vs no	2.9 (0.8–12.3)	0.1178	2.3 (0.6–10.4)	0.2396
^a^Change in ctDNA: ≥80% vs < 80%	8.5 (1.4–163)	0.0150	7.4 (1.2–144)	0.0276

*CEA* carcinoembryonic antigen, *ctDNA* circulating tumour DNA, *NAC* neoadjuvant chemotherapy, *CRT* chemoradiotherapy, *SRT* short-course radiotherapy.

^a^Change in ctDNA, 1—after/before the ratio of ctDNA (%).

### Association between postoperative ctDNA and clinical outcome

**Fig. 3:**
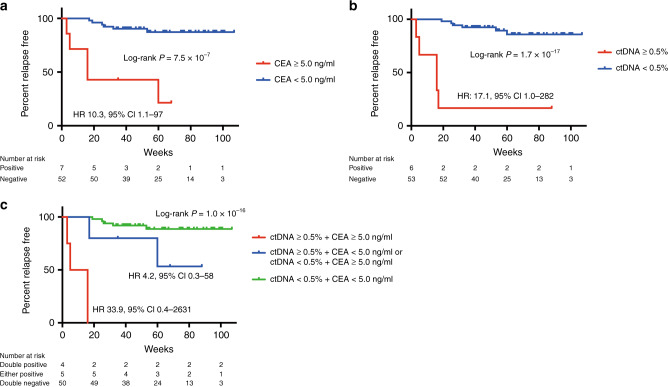
Kaplan–Meier estimates of recurrence-free survival for postoperative CEA, postoperative ctDNA levels and the combined effect. **a** Comparison of patients with normal levels of postoperative CEA (<5.0 ng/ml) and those with high levels (≥5.0 ng/ml). **b** Comparison of patients with low levels of postoperative ctDNA (<0.5%) and those with high levels (≥0.5%). **c** Effect of combined analysis of postoperative CEA and ctDNA levels.

We next investigated the clinical significance of postoperative ctDNA as a prognostic marker of clinical outcome after radical operation in 59 patients with LARC (Supplementary Fig. 1). In the univariate Cox proportional hazard analysis, postoperative CEA, ypT factor, lymphovascular invasion and postoperative ctDNA were considered to be significantly associated with recurrence-free survival after surgery (Table 3). In the multivariate analysis, postoperative CEA and ctDNA levels still remained independent prognostic factors of postoperative recurrence after adjusting for six parameters used in the univariate analysis (*P* = 0.0105; adjusted HR, 6.9; 95% CI, 1.6–29 for postoperative CEA and *P* = 0.0127; adjusted HR, 7.7; 95% CI, 1.6–42 for postoperative ctDNA, Table 3). Kaplan–Meier estimates indicated significantly different recurrence-free survival for patients with higher postoperative CEA (≥5 ng/ml) and higher postoperative ctDNA (≥0.5%) (Log-rank *P* = 7.5 × 10^–7^ for CEA, Log-rank *P* = 1.7 × 10^–17^ for ctDNA, Fig. 3). Furthermore, a combined analysis of postoperative CEA and ctDNA revealed cumulative effects on recurrence-free survival (Log-rank *P* = 1.0 × 10^–16^). The adjusted HR for risk of recurrence computed for patients carrying risk factors increased from 4.2-fold (either higher CEA or ctDNA) to 33.9-fold (both of them) compared with those without any risk factors (Fig. 3).

**Table 3:** Cox proportional hazard model for recurrence-free survival.

Variables	Univariate analysis		Multivariate analysis	
	HR (95% CI)	*P* value	HR (95% CI)	*P* value
Gender: male vs female	2.9 (0.5–53)	0.2457		
Postoperative CEA: ≥5.0 ng/ml vs < 5.0 ng/ml	11 (3.0–36)	0.0006	6.9 (1.6–29)	0.0105
ypT factor: T3–4 vs T0–2	5.6 (1.4–37)	0.0112	1.1 (0.1–12.3)	0.9279
ypN factor: positive vs negative	2.7 (0.8–10)	0.1091	1.4 (0.3–6.7)	0.6332
Lymphovascular invasion: positive vs negative	10 (2.6–67)	0.0005	5.3 (0.6–79)	0.1276
Tumour regression grade (Dworak): grade 1–2 vs grade 3–4	2.9 (0.8–19)	0.1272	2.0 (0.2–18.9)	0.5236
NAC with CRT/SRT: yes vs no	1.2 (0.3–3.3)	0.9946		
Postoperative ctDNA: ≥0.5% vs < 0.5%	20 (5.6–72)	<0.0001	7.7 (1.6–42)	0.0127

*CEA* carcinoembryonic antigen, *NAC* neoadjuvant chemotherapy, *CRT* chemoradiotherapy, *SRT* short-course RT, *ctDNA* circulating tumour DNA.

## Discussion

This study represents the first association between response to preoperative therapy and the results of serial ctDNA analysis for patients with LARC. In this study, we show that the change in ctDNA measured in consecutive samples could act as an indicator of response to preoperative therapy (pCR) in patients with LARC. Through clinical and genetic analyses, we show that postoperative ctDNA and CEA were significantly associated with recurrence-free survival in 59 Japanese patients with LARC after preoperative therapy and radical operation. Furthermore, a combined analysis of postoperative ctDNA with postoperative CEA levels revealed that the number of risk factors (0, 1 or 2 factors) has a cumulative effect on the rate of recurrence-free survival in patients with LARC. These findings may help physicians to select the best treatment strategy for patients with LARC after preoperative therapy (i.e., surgery vs the watch-and-wait approach) and choose an optimal adjuvant therapy based on the results of the postoperative ctDNA and CEA levels.

Numerous studies have sought to identify clinically useful predictors of pCR after preoperative radiation therapy and/or chemotherapy for patients with LARC.^4,8–11,20,26,27^ Several parameters have been reported as possible predictors of the response to preoperative CRT in patients with LARC, including CEA levels before CRT, the distance of the tumour from the anal verge, tumour size, clinical lymph node metastasis and the interval between CRT and surgery.^4,8,9,11^ Habr-Gama et al. suggested that a strict definition of the clinical and endoscopic findings of patients with cCR after preoperative CRT could be used to select for patients who could be managed with a watch-and-wait strategy.^28^ Others, however, have reported that endoscopic evaluation for the response to preoperative CRT has low sensitivity to detect pCR.^12,14,22,28^ Although studies have reported an association between clinical outcomes and cfDNA or ctDNA among patients receiving CRT for LARC,^17,29,30^ no study has reported the positive correlation between pCR and cfDNA or ctDNA after CRT. Intensified regimens, such as NAC with CRT, might have had an impact on the response to preoperative therapy. Indeed, in the univariate analysis, NAC with CRT/SRT showed a trend towards an increased response to preoperative therapy. However, the multivariate logistic regression analysis showed only change in ctDNA as an independent predictor for treatment response (Table 2). The present study is the first to identify a change in ctDNA as a promising predictor of response to preoperative chemotherapy and/or radiation therapy in patients with LARC. Although the positive and negative predictive values were 33.3% (11 responders among 33 patients with the change in ctDNA ≥ 80%) and 94.4% (17 non-responders among 18 patients with the change in ctDNA < 80%), respectively, the positive predictive value increased to 54.5% (6 responders among 11 patients with endoscopic CR and a change in ctDNA ≥ 80%) when the change in ctDNA data was combined with endoscopic findings (Supplementary Fig. 5). Hence, a combinatorial analysis of the change in ctDNA with clinical factors, including endoscopic findings, might help to identify and select for patients who do not need immediate surgical management; further validation studies are needed to verify the clinical utility of this proposal.

In our study, the number of patients with mutations decreased after preoperative therapy (Supplementary Figs. 3 and 4), and this reduction was noted for all genes, presumably because tumour shrinkage after preoperative therapy reduced the amount of ctDNA available for analysis. The number of patients with *TP53* mutations—the most frequently detected in this study—was significantly lower after preoperative therapy amongst responders (*P* = 0.026) but not amongst non-responders (Supplementary Table 1). *TP53* is one of the best-studied tumour-suppressor genes^31^—referred to as the guardian of the genome—and represents a key regulator of cellular growth control.^32^ p53 plays a critical role in regulating DNA repair and apoptosis in response to radiation, and *TP53* mutations are reported to decrease radiation-induced apoptosis in several types of cancers.^33,34^ These lines of evidence suggest that chemoradiation therapy provides a selective pressure for the expansion of *TP53*-mutant cells in residual tumours; further analysis using a larger number of patients and comparing the mutational status of tissues and plasma is required to verify the above hypothesis.^35^

There were several limitations in this study. First, because the number of recruited patients we assessed was small and the follow-up period was short, there were too few events to correct for potential confounding factors in the multivariate analyses. Second, the preoperative therapy regimen was not completely standardised, and patients underwent different approaches, based on different risk profiles. Third, tumour biopsy sequencing was not performed in our study. However, mutations in cfDNA, which are not detected in tumour biopsies, may comprise a subset of alterations that reflect ongoing tumour evolution and heterogeneity not captured in the small and usually anatomically constrained biopsy. Moreover, the proportion of patients with positive ctDNA at baseline was low, and the frequencies of mutated genes in plasma ctDNA samples from patients with LARC in our study were inconsistent with those measured from DNA tissue samples recorded in The Cancer Genome Atlas database.^36,37^ For example, the frequency of *APC* mutations in colorectal cancer tissue samples is reported to be ~80%,^36,38^ whereas we found a mutation frequency of only 17.1%. The differences in the frequencies could be partially due to an insufficient coverage of mutation detection for the *APC* gene in our ctDNA study. Further technical improvement in the gene panel would increase the detection accuracy among patients with LARC.

In conclusion, we show that serial ctDNA analysis is applicable for the prediction of treatment response among patients with LARC who undergo preoperative therapy. Our study also shows that postoperative ctDNA may offer a strong indicator of clinical outcome after radical operation for patients with LARC. Moreover, we demonstrate a cumulative effect of combining postoperative ctDNA with postoperative CEA levels as prognostic markers of recurrence-free survival among patients with LARC who are treated surgically. Our findings provide new insight into precision medicine for patients with LARC. To improve the quality of life of patients with LARC, future studies should integrate ctDNA testing of hundreds of cancer-related genes with large amounts of clinical data to improve patient selection and management.

## Supplementary information

Supplementary Table 1

Supplementary Material

## Data Availability

The datasets generated and/or analysed during the current study are available from the corresponding author on reasonable request.
