# Supplementary material for: Serial circulating tumour DNA analysis for locally advanced rectal cancer treated with preoperative therapy: prediction of pathological response and postoperative recurrence
Source: Br J Cancer. 2020 Jun 22;123(5):803–10. doi: 10.1038/s41416-020-0941-4 (PMC7462982; doi:10.1038/s41416-020-0941-4)
Supplement: Supplementary file 2 — Supplementary Material [file 41416_2020_941_MOESM2_ESM.docx]

**
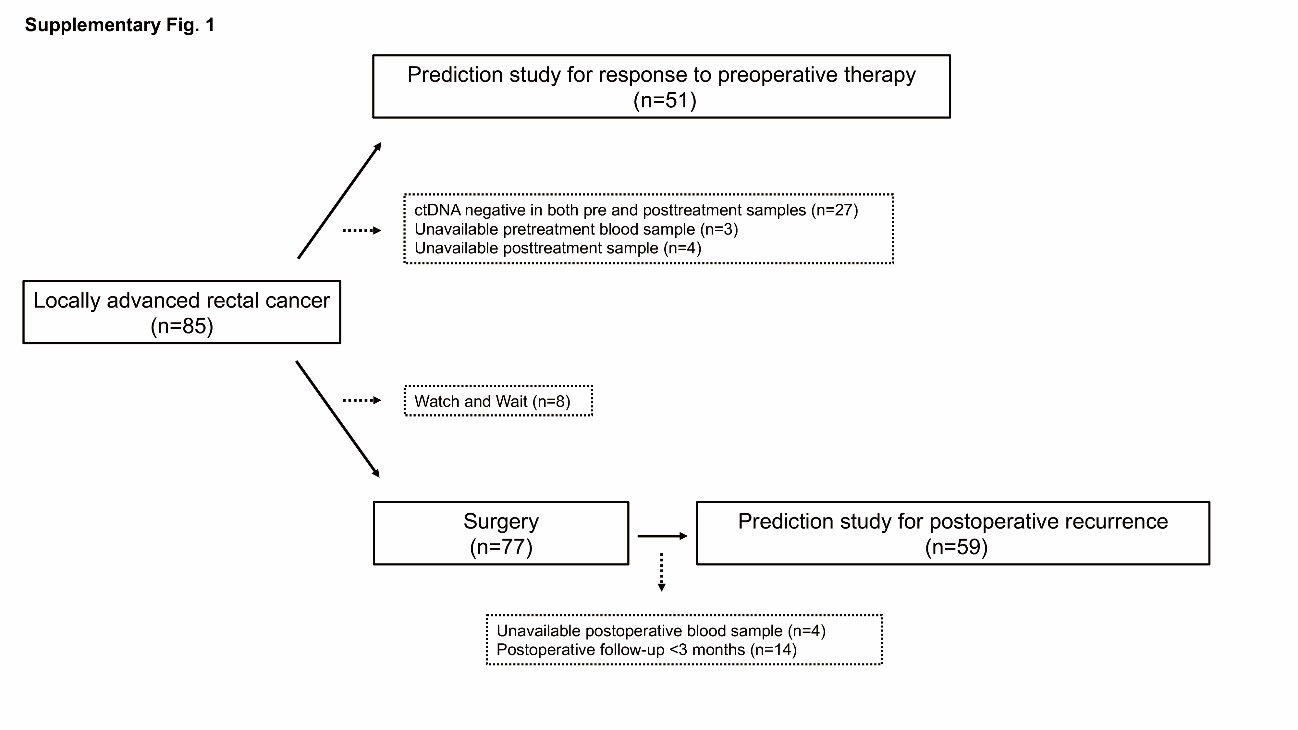
**

**Supplementary Figure 1. A schematic illustration of the study design.**

Blood samples were collected before and after preoperative therapy, and after surgery. Eighty-five patients were treated with preoperative therapy. Of these, 77 patients received radical operation and 8 patients were managed by the watch-and-wait approach. Of the 77 patients treated surgically, postoperative plasma samples were available for 59 patients. Samples from 51 patients were analyzed for response to preoperative therapy, and samples from all 59 patients were analyzed for the prediction of postoperative recurrence.

**
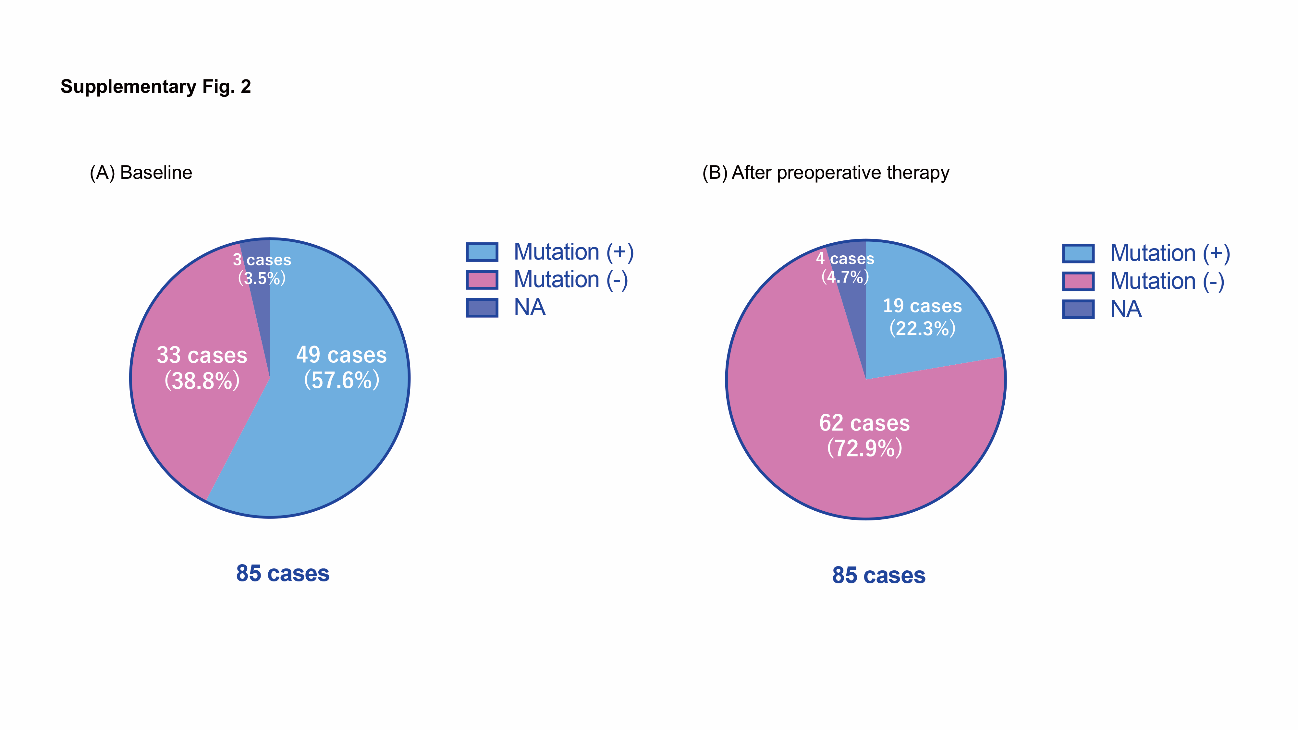
**

**Supplementary Figure 2. Somatic mutations detected in plasma before and after preoperative therapy.**

Mutations were detected in 57.6% and 22.3% of patients before and after preoperative therapy, respectively. The proportion of mutations was significantly decreased after preoperative therapy (*P* < 0.0001). NA, not available (no blood sampling).

**
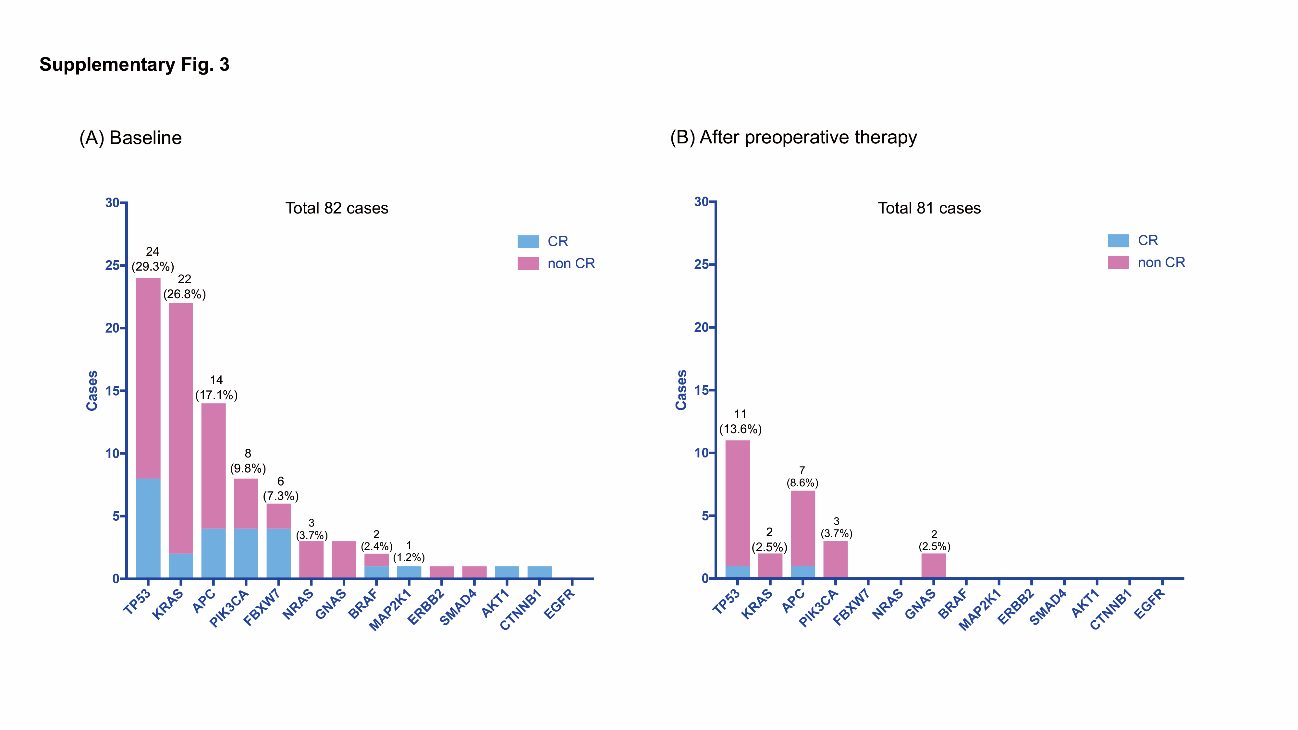
**

**Supplementary Figure 3. Number of patients with mutations before and after preoperative therapy.**

(A) 29.3%, 26.8%, and 17.1% of patients had mutations in *TP*53, *KRAS*, and *APC*, respectively. (B) *TP53* and *APC* were the most frequently mutated genes after preoperative therapy, detected in 13.6% and 8.6% of patients respectively. CR, complete response.

**
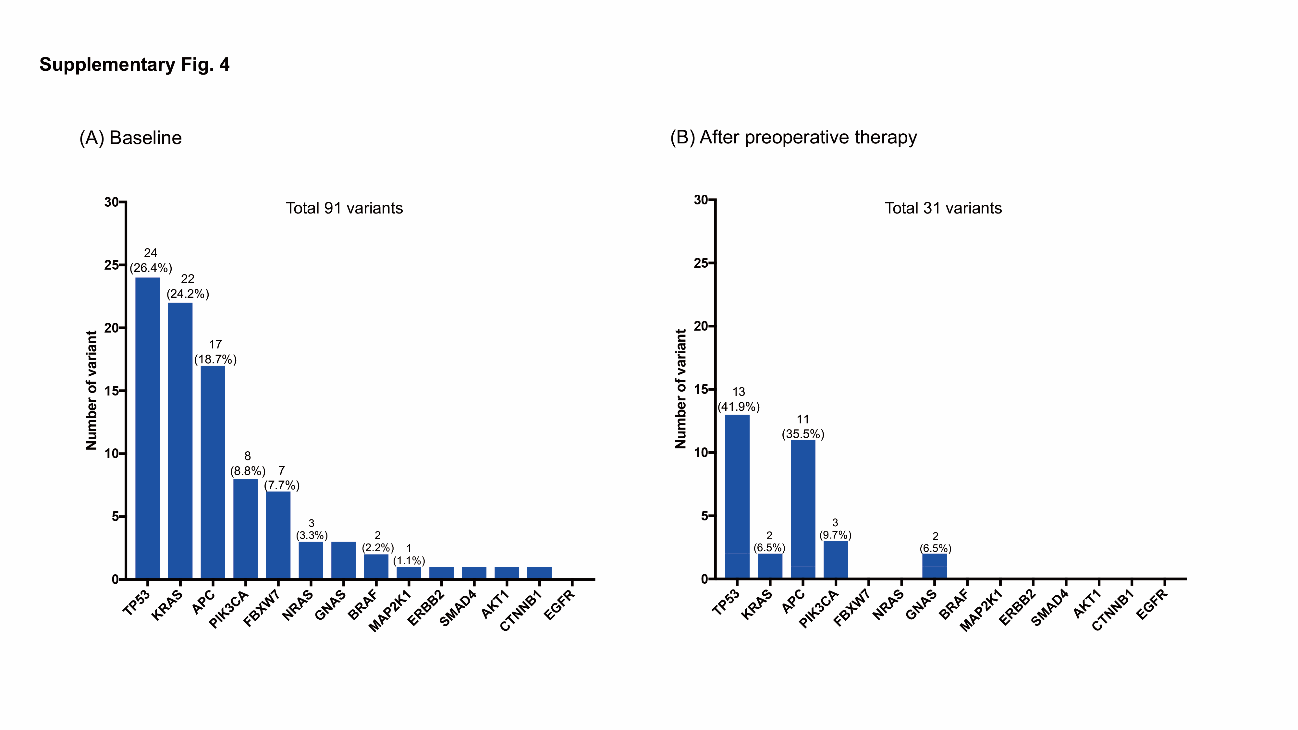
**

**Supplementary Figure 4. Number of mutations detected before and after preoperative therapy.**

(A) 24, 22, and 17 mutations were detected in *TP53*, *KRAS*, and *APC* at baseline, respectively. (B) 13 and 11 mutations were detected in *TP53* and *APC* after preoperative therapy, respectively.

**
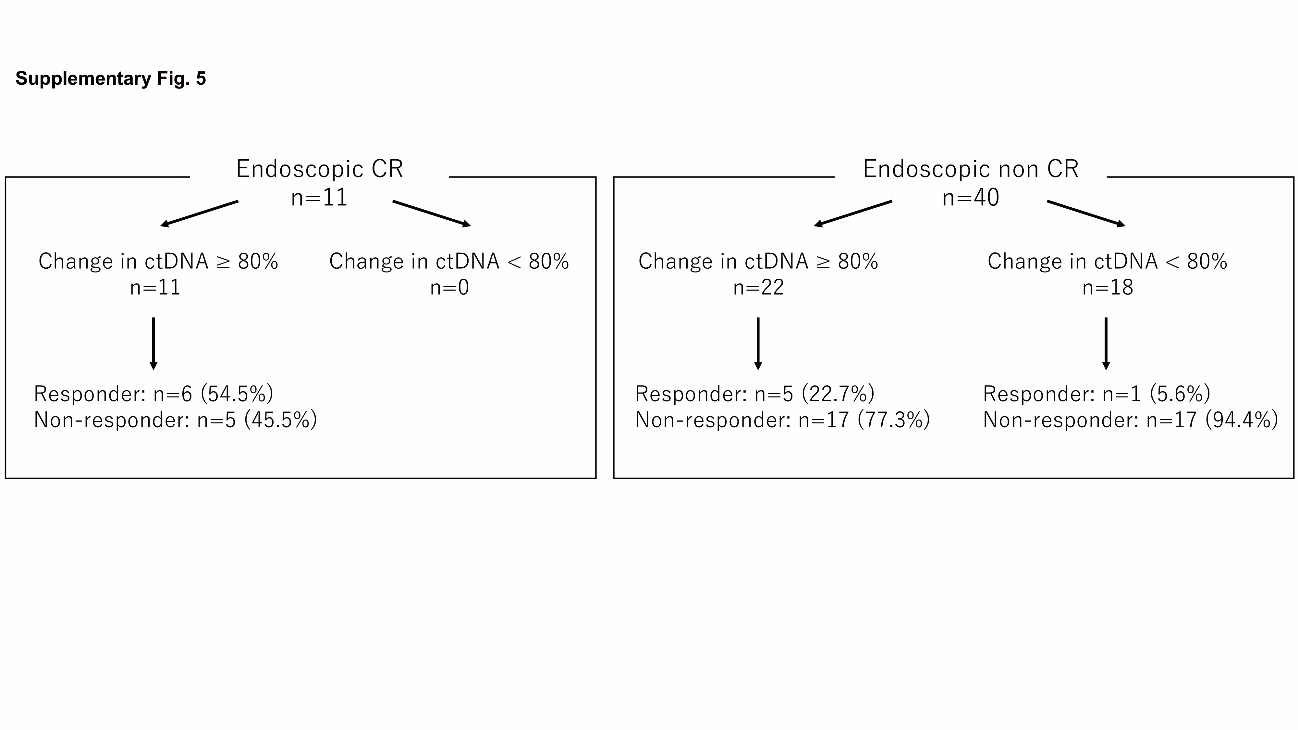
**

**Supplementary Figure 5. Response stratification using endoscopic findings and ctDNA response.**

CR, complete response; ctDNA, circulating tumor DNA.
